# Morphological and Molecular Identification of *Anisakis* spp. (Nematoda: Anisakidae) in Commercial Fish from the Canary Islands Coast (Spain): Epidemiological Data

**DOI:** 10.3390/ani12192634

**Published:** 2022-09-30

**Authors:** Natalia Martin-Carrillo, Katherine García-Livia, Edgar Baz-González, Néstor Abreu-Acosta, Roberto Dorta-Guerra, Basilio Valladares, Pilar Foronda

**Affiliations:** 1 Instituto Universitario de Enfermedades Tropicales y Salud Pública de Canarias, Universidad de La Laguna, Avda. Astrofísico F. Sánchez s/n, 38203 San Cristóbal de La Laguna, Canary Islands, Spain; 2 Department Obstetricia y Ginecología, Pediatría, Medicina Preventiva y Salud Pública, Toxicología, Medicina Legal y Forense y Parasitología, Universidad de La Laguna, Avda. Astrofísico F. Sánchez s/n, 38203 San Cristóbal de La Laguna, Canary Islands, Spain; 3 Nertalab S.L. José Rodríguez Mouré, 4, Bajo, 38008 Santa Cruz de Tenerife, Canary Islands, Spain; 4 Department Matemáticas, Estadística e IO, Universidad de La Laguna, 38206 La Laguna, Canary Islands, Spain

**Keywords:** *Anisakis simplex* (s.s.), *Anisakis pegreffii*, Anisakis typica, *Anisakis physeteris*, *Anisakis nascettii*, Canary Islands, fish-borne zoonoses, anisakiasis, seafood safety

## Abstract

**Simple Summary:**

Anisakidae is a family in the phylum Nematode, which is probably the most prevalent family of parasites found in marine mammals. These nematodes are colonizers of the digestive system of marine vertebrates that use different crustaceans and fish species as intermediate or paratenic hosts and humans as accidental hosts. Human anisakidosis, an infection caused by some species of the family Anisakidae, occurs when shellfish, particularly fish, contaminated with the infectious stage (third-stage larvae [L3]) of this parasite are consumed raw or undercooked. Data on the species of *Anisakis* found in fish in the waters of the Canary Islands and epidemiological data in general from this archipelago on this parasitic nematode are scarce, so the aim of this study was to provide these data on fish with an interest in consumption in the Canary Archipelago. Five species of *Anisakis* were identified, of which two have health relevance to humans. These findings are valuable for the safe consumption of fish by the population and for the knowledge of health authorities when it comes to a better diagnosis in hospitals and for future epidemiological and biomedical research.

**Abstract:**

The study aimed to perform the molecular identification of *Anisakis* larvae in commercial fish from the coast of the Canary Islands and to provide data on their infection level for the host and the species of this nematode parasite that we could find in several species of commercial interest in the Canary Archipelago. Fish specimens (n = 172) from the Canary coasts were examined for parasites. In total, 495 larvae were identified; PCR was carried out for the entire ITS rDNA and cox2 mtDNA region, obtaining sixteen sequences for the entire ITS rDNA region and fifteen for the cox2 mtDNA, this being the first contribution of nucleotide sequences of *Anisakis* species of fish caught from the Canary Islands. An overall prevalence of 25% was obtained in the fish analyzed, and five species of *Anisakis* were identified, these being *Anisakis simplex* (s.s.), *Anisakis pegreffii, Anisakis physeteris*, *Anisakis nascettii* and *Anisakis typica* and the hybrid *Anisakis simplex* x *Anisakis pegreffii*. The results obtained in this study have relevance for public health, since the pathology will depend on the species of *Anisakis,* so it is important to know the health status of fish in the waters of the Canary Islands to assure a safer consumption and take adequate measures, in addition to the provision of epidemiological data.

## 1. Introduction

Anisakids are parasitic nematodes belonging to the Anisakidae family and subfamily Anisakinae. This family includes, among others, the genera of health relevance, *Anisakis*, *Pseudoterranova* and *Contracaecum* [1]. These parasites have an indirect life cycle; in the case of *Anisakis* species, it involves cetaceans as final hosts, zooplankton as intermediate hosts and fish and cephalopods as intermediate or paratenic hosts [2]. Humans act as accidental hosts, acquiring infection through consumption of raw, cold-smoked, marinated, salted or undercooked fish and squids infected with third-stage larvae of *Anisakis* species. Anisakid nematodes are known to have low host specificity in the larval stage, and, in fact, more than 200 fish and 250 cephalopods species have been reported to harbor anisakids nematodes [3]. The presence of *Anisakis* spp. in fisheries products is of enormous importance to consumers and food safety authorities, and fisheries and food industries, due to the associated disease and possible loss of value. 

Anisakidosis is one of the most important fish-borne zoonoses worldwide [4], caused by nematodes of the Anisakidae family (genera *Anisakis*, *Pseudoterranova* and *Contracaecum*). The family has the potential to affect human health, although not all the anisakid species cause diseases in humans [5]. The term “anisakiasis” applies only to the disease caused by *Anisakis* species [5]. At present, two species of the genus *Anisakis* are reported as the causative agent of infections in humans, *Anisakis simplex,* sensu stricto (s.s), and *Anisakis pegreffii* [6,7], with the first two being responsible for the most reported cases of human infection. Currently, it is estimated that the total number of worldwide anisakidosis may be over 76,000, and half of new cases are noted in Europe, especially in Spain and Italy [8]. Cases have been reported from the five continents: in Europe, for example, in Spain [9,10], the Netherlands [11], Germany [12], Italy [13,14], Croatia [15], France [16] and the United Kingdom [17]; in Africa, in Egypt [18]; in the Americas, in Peru [19], Hawaii [20] and Canada [21]; as well as in Asia, specifically in Japan [4,22], Taiwan [23], Korea [24] and in New Zealand [25].

According to the European Food Safety Authority [26], anisakid nematodes constitute the most important “biological hazard” in seafood products. Although the first case of anisakid infection was described by Luckart in Greenland in 1876 [27], the disease was more widely described in the 1950s and 1960s, when epidemics of anisakidosis occurred in the Netherlands [28]. The clinical manifestations and symptoms of anisakidosis depend on several factors, such as the immune system of each person infected, and on the anisakid species involved in infection. Symptoms of acute anisakidosis include severe abdominal pain, nausea and vomiting. Some of these symptoms closely mimic peptic ulcer, appendicitis or peritonitis, with the most concerning presentation being allergic sensitization, which is usually serious and ranges from urticaria to anaphylactic shock [29]. To date, the treatment for gastric anisakisis usually involves endoscopic removal of the worm, while patients with intestinal disease may require surgical resection. There are some reports of albendazole or thiabendazole treatment in isolated cases of human anisakiasis [30,31,32]. Nevertheless, the best protection against anisakiasis is to educate consumers about the dangers of eating raw fish and to recommend avoiding the consumption of raw or inadequately thermally treated marine fish or cephalopods. 

Since the discovery of the diseases, the prevalence of anisakidosis cases has increased markedly worldwide. This increase may be due to several factors; on the one hand, previously, many cases of gastric anisakidosis were underdiagnosed or misdiagnosed, and currently, with the use of techniques, as for, example with endoscopy, this diagnosis has been improved. On the other hand, the culinary habits have changed around the world, and in regions where the consumption of raw fish or fish prepared with techniques that do not kill the parasite was not so common, now, its consumption has increased, especially in many Western countries, with an increased risk of exposure to the parasites [33]. The EFSA recommends that research on parasites in fishery products involved in public health be continued [26]. In Spain, to ensure compliance with food hygiene rules by the operators concerned, Regulation (EC) No 854/2004 [34] lays down specific rules for the organization of official controls on products of animal origin intended for human consumption. These controls are carried out through control procedures or programs implemented by the autonomous communities.

Due to the scarcity of data in the Canary Islands on the epidemiology of *Anisakis* spp. and its prevalence in the different species of fish on the Canary coast, and the possible risk associated with the consumption of fish, it is necessary to clearly understand the distribution of the anisakids species and their hosts. The purpose of this study was to identify the anisakids larvae present in different fish species of economic importance collected from markets and caught in the Canary Coast, using a genetic-molecular approach to improve the epidemiological data of species of anisakids present in fish from the waters of the Canary Islands.

## 2. Materials and Methods

Between October 2020 and December 2021, 172 specimens of 11 commercial fish species caught in the Canary coast (13°23′–18°8′ W and 27°37′–29°24′ N), specifically in zone FAO 34, described and designated by the Food and Agriculture Organization, were collected from different markets in Tenerife island and examined: frigate tunas (*Auxis thazard*; n = 14), white seabream (*Diplodus sargus sargus*; n = 2), European hake (*Merluccius merluccius*; n = 27), striped red mullet (*Mullus surmuletus*; n = 6), common pandora (*Pagellus erythinus*; n = 11), sardinelle (*Sardinella aurita*; n = 21), salema (*Sarpa salpa*; n = 5), Atlantic chub mackerel (*Scomber colias*; n = 30), mackerel (*Scomber scombrus;* n = 16), black tail comber (*Serranus atricauda*; n = 6) and blue jack mackerel (*Trachurus picturatus*; n = 34). All the collected specimens were measured for their length (total length, TL) and weight (body weight, TW) before necropsy and analysis. 

After measurement, the fish were dissected and examined for anisakids. Briefly, peritoneal cavity and digestive tract of fish samples were examined after making an incision along the ventral line from the anus to the mouth. The viscera and muscles were isolated in physiological saline solution and examined under Leica M80 stereo microscope (Leica Mikrosysteme Vertrieb GmbH, Wetzlar, Germany) for the presence of nematode larvae. The collected anisakid larvae were washed in saline solution, then fixed and preserved in 70° ethanol. A random selection of these larvae was made for the molecular analysis: eight from *A. thazard*, thirty from *M. mercluccius*, seven from *S. colias* and twelve from *S. scombrus*. These specimens were cut in three portions; the central part was reserved for molecular identification, and the anterior and posterior parts were cleared and mounted in lactophenol between the glass slide and coverslip for morphological identification. The morphologic features of the larvae were analyzed according to the available diagnostic keys [35,36,37]. The microphotographs were taken with the Leica DM750 microscope model ICC50 HD, (Leica Microsystems, Heerbrugg, Switzerland), and the measurement software used was the Leica application suite (LAS) version 4.112.0 (License-Number. LAS32696, Leica Microsystems, Heerbrugg, Switzerland). The characterization was mainly based on the presence/absence of the mucron, the anatomy of the anterior part of the digestive tract, the presence/absence of an apical tooth and lips, the caudal anatomy and the position of the excretory pore. 

The results are presented as means ± standard deviations (SD) for continuous data and proportions (prevalence) for categorical data. Epidemiological indices of prevalence (P%), mean abundance (MA) and mean intensity (MI) of infection were calculated according to Ref [38]. The 95% confidence intervals for prevalence using the approximate or exact method, as appropriate, were included. For mean abundance and mean intensity, a 95% confidence interval was provided based on normal theory only if the number of infested individuals was large enough (≥30) in the sample. Otherwise, the bias-corrected and accelerated approach (BCa) bootstrap confidence interval of Ref [39] was used. For the length and weight of fish, a 95% confidence interval based on normal distribution was provided when the assumption of normality was met, as assessed by the Q-Q Plots and the Shapiro-Wilk test. When normal distribution was not achieved, a BCa confidence interval was used. Data analyses were carried out using the IBM^®^ SPSS^®^ version 25 (IBM Corporation, Armonk, NY, USA) and R 4.0.2, (Vienna, Austria) [40] statistical software.

In order to obtain a molecular identification of the collected anisakids larvae, the total genomic DNA was extracted following Ref [41], where each central part of the parasite found reserved in 70% ethanol was ground in a 1.5 mL tube containing 250 µL of lysis buffer (30 mM Tris-HCl pH 8.0, 10 mM EDTA, 0.4% SDS) and 3 µL of proteinase K (20 ng/µL) and then incubated at 56 °C overnight. Afterward, 250 µL of 4M NH_4_Ac was added and mixed thoroughly, followed by 30 min incubation at room temperature. Afterward, the samples were centrifuged at 13,000 rpm for 10 min, and the supernatant was transferred to another tube and submitted to centrifugation with absolute ethanol and 70% ethanol in two consecutive steps. The DNA extraction procedure was checked using DeNovix DS-11+ Spectrophotometer (Wilmington, USA). The entire region of the nuclear ribosomal internal transcribed spacer (ITS rDNA) was amplified using primers NC5 (5′-GTAGGTGAACCTGCGGAAGGATCATT-3′) and NC2 (5′-TTAGTTTCTTTTCCTCCGCT-3′) [42]. On the other hand, the mitochondrial cytochrome c oxidase subunit II gene (cox2 mtDNA) was amplified using primers 211F (5′-TTTTCTAGTTATATAGATTGRTTYAT-3′) and 210R (5′-CACCAACTCTTAAAATTATC-3′) [43]. The PCR amplification contained 1X Buffer (Bioline, London, UK), 0.2 mM of each dNTP (Bioline, London, UK), 1 µM of each primer, 1U of Taq DNA polymerase (Bioline, London, UK), 1.5 mM MgCl_2_ (Bioline, London, UK) and 20–30 ng of total genomic DNA in a total volume of 50 µL. The amplification was conducted with an XP Cycler (Bioer Technology Co., Hi-tech, Hangzhou, China) using the following parameters: 3 min at 94 °C followed by 35 cycles of denaturation at 94 °C for 30 s, annealing at 55 °C for 30 s and extension at 72 °C for 30 s, with a final extra extension step at 72 °C for 5 min. The PCR products were detected by electrophoresis of 1 μL of amplified DNA using 1.2% agarose gel at 100 V for 45 min.

The PCR products for the entire ITS rDNA and cox2 mtDNA region were sequenced in Macrogen (Madrid, Spain) with the Sanger sequencing method. The analysis of the sequences was carried out with MEGA X (Molecular Evolutionary Genetic Analysis) software (Hachioji, Japan) [44], using the multiple alignment program ClustalW included in MEGA X, and minor corrections were made by hand. Sequences were compared with the available published sequences in GenBank by the BLAST (blastn) program. The molecular identification of the entire ITS rDNA and cox2 mtDNA region was achieved by phylogenetic analysis trough the neighbor-joining distance method (NJ) with the p-distance model [45] and maximum-likelihood (ML) method with the Tamura-Nei model [46], both with at least 1000 bootstrap replications in MEGA X using the sequence *Contracaecum osculatum* (Acc. Number.: MT288528) as the outgroup.

## 3. Results

The presence of *Anisakis* spp. larvae was detected in 25% of the fish analyzed, four of the eleven species being positive: *A. thazard* (21.43%; 3/14), *M. mercluccius* (85.18%; 23/27), *S. colias* (26.6%; 8/30) and *S. scombrus* (62.5%; 10/16). The overall results obtained for the 172 examined fish are reported in Table 1.

**Figure 1:**
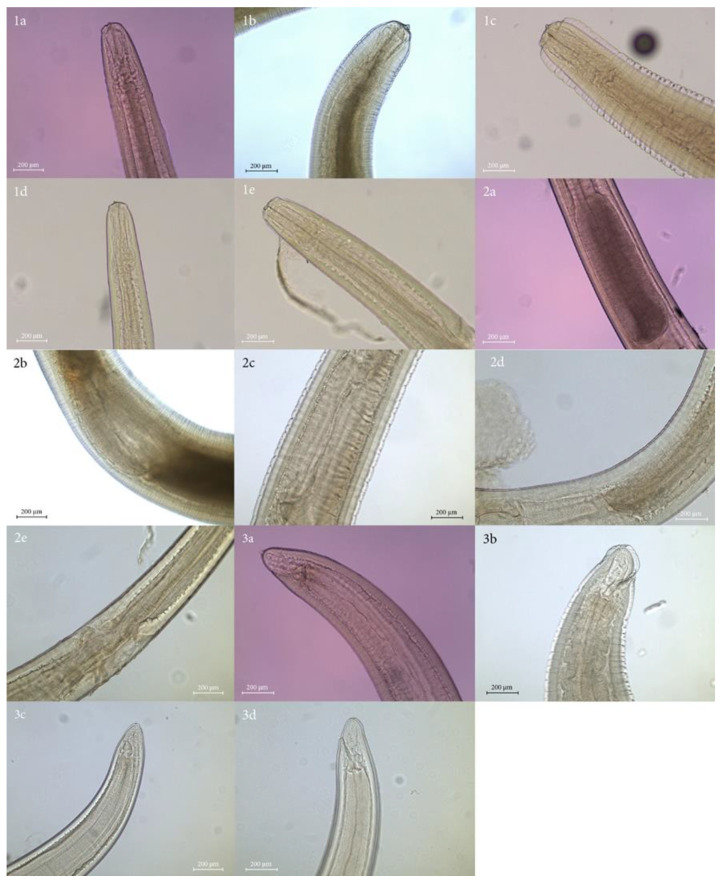
Microscopic images of third-stage larvae of *Anisakis* species found in this study (magnification 20×). (**1a**–**1e**), anterior regions of *Anisakis pegreffii, Anisakis physeteris, Anisakis nascettii, Anisakis simplex* (s.s.) and *Anisakis typica*, respectively. (**2a**–**2e**), middle part of *A. pegreffii, A. physeteris, A. nascettii, A. simplex* (s.s.) and *A. typica*, respectively. (**3a**–**3d**), posterior parts of *A. pegreffii, A. nascettii, A. simplex* (s.s.) and *A. typica*, respectively. All images correspond to larval Type I, with the exception of images (**1b**) and (**2b**), which correspond to larval Type II.

A total of 495 larvae were collected and firstly assigned morphologically [35,36,37] to the larval morphotype-Type I (n = 489) and Type II (n = 6) (Figure 1). Type I larvae had an elongated ventricle and a mucron at the caudal end, and Type II larvae had a shorter ventricle and no mucron. The larvae identified as Type I presented differences in the ventricular and intestinal junction; some showed an oblique junction, and others had a straight junction. In addition, the larvae of Type I had a short and rounded tail, while the larvae of Type II had a more elongated tail.

From the random selection of larvae used for molecular identification, sixteen sequences were obtained for the entire ITS rDNA region and fifteen for the cox2 mtDNA gene. Nucleotide sequences obtained in this work were submitted to the GenBank database under the accession numbers OM328159-OM328168, OM328178-OM328183 for the entire ITS rDNA region and OM417311-OM417325 for cox2 mtDNA.

The amplification of the entire ITS rDNA region and cox2 mtDNA produced a fragment of approximately 1200 bp and 672 bp, respectively. The blast search showed that the sequences identified in this study matched with previously reported nucleotide sequences of the *Anisakis* species: *A. simplex* (s.s.), *A. pegreffii*, the hybrid *Anisakis simplex* × *Anisakis pegreffii*, *Anisakis nascettii*, *Anisakis physeteris* and *Anisakis typica*. The list of hosts and species of *Anisakis* identified in them is shown in Table 2 (Table S1).

The alignments of 1026 bp and 553 bp for the entire ITS rDNA region and cox2 mtDNA, respectively, were used for phylogeny. For the entire ITS rDNA region, the sequences with Acc. number OM328163 and OM328159 were not taken into account for the quality of the chromatogram. 

**Figure 2:**
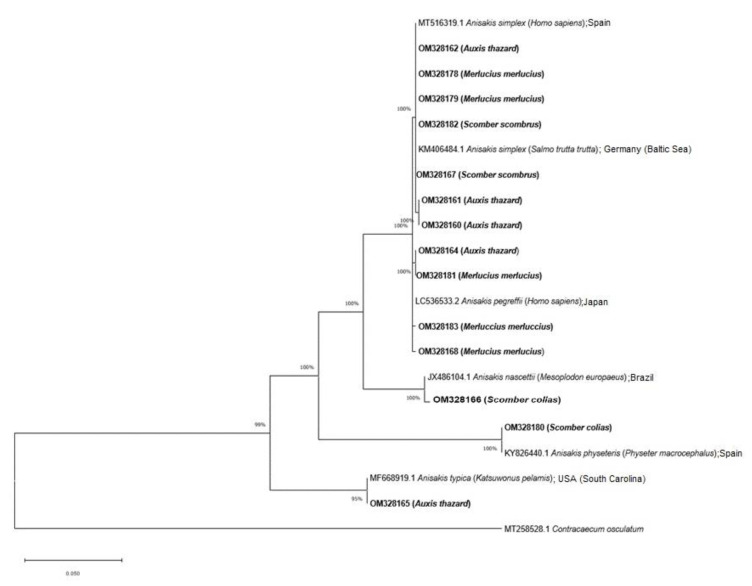
Phylogenetic analysis using the neighbor-joining method with p-distance and 1000 bootstrap replications based on the entire ITS rDNA region. Sequences exploring the relationships among *Anisakis* spp., including the nucleotide sequences obtained in this study (shown in bold). *Contracaecum osculatum* was used as an outgroup.

**Figure 3:**
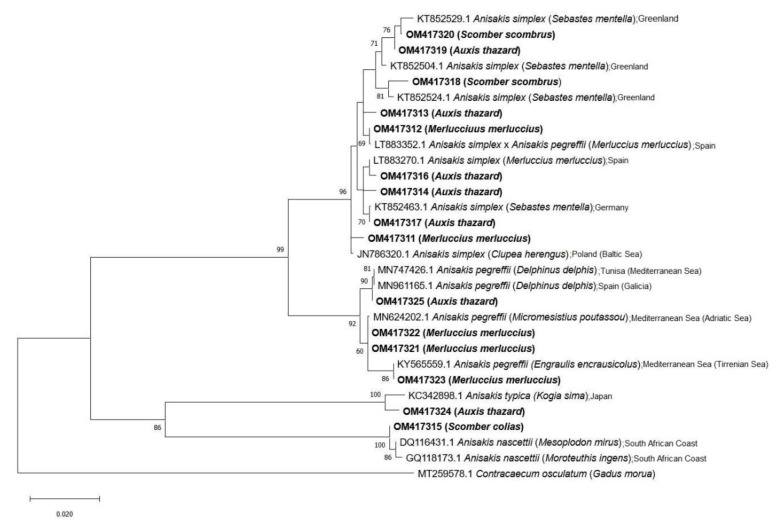
Phylogenetic analysis using the maximum likelihood method with p-distance and 1000 bootstrap replications based on the cox2 mtDNA. Sequences exploring the relationships among *Anisakis* spp., including the nucleotide sequences obtained in this study (shown in bold). *Contracaecum osculatum* was used as an outgroup.

The phylogeny analyses confirm the identity of the *Anisakis* species observed by BLAST. The trees based on the entire ITS rDNA region and cox2 mtDNA gene show a clade, including the species complex *Anisakis* composed of *A. simplex* (s.s.), *A. pegreffii* and also the hybrid *A. simplex* × *A. pegreffii* with a bootstrap value of 100% and 99%, respectively. These species were obtained from *A. thazard*, *M. merluccius* and *S. scombrus* for *A. simplex* (s.s.), and from *A. thazard* and *M. merluccius* for *A. pegreffii* and the hybrid *A. simplex* × *A. pegreffii*. On the other hand, the species *A. nascettii* found in *S. colias* forms a distinct clade with 100% bootstrap. *Anisakis physeteris* found in *S. colias,* is included in the other clade with 100% bootstrap for the entire ITS rDNA region. Finally, *A. typica* identified in *A. thazard* is included in the clade of this species with 95% and 86% bootstrap, respectively (Figure 2 and Figure 3). 

## 4. Discussion

The oceanographic characteristics of the Canary Islands are influenced directly by the Canary current (descending branch of the Gulf Stream), which flows south-southwest and transports cold water from the northernmost latitudes and to their proximity to the African continent, where an outcropping of deep water of an east-west direction is generated, which generates a strong productive development [47,48]. In turn, the islands function as barriers to atmospheric and oceanic flows due to the presence of trade winds and the Canary Islands current that induces the formation of structures such as eddies between the islands and warm trails to leeward [49,50], which influence and delimit the possible areas of important production and accumulation of plankton biomass, while it could accumulate or disperse the larvae of fish and zooplankton [51,52,53,54,55]. These factors, in addition to the orography of the archipelago, provide the optimal conditions to host in its water an enormous marine diversity, 5300 marine species being present in the Canary Islands, and of these, 500 fish and 80 cetaceans species [56]. Specifically, this number of cetaceans constitutes 30% of whales and dolphins known today, making the Canary Islands one of the places in the world with the greatest diversity of cetaceans [50]. For food safety, the parasitological study of the anisakids species in shellfish, fish and cephalopods is limited due to the increasing numbers of fish and crustacean hosts for *Anisakis* spp. [26]. Almost three decades ago, the most common hosts of *A. simplex* (s.s.) were spotted chub mackerel (*Scomber japonicus*) and Japanese flying squid (*Todarores pacificus*) [57]. Furthermore, Abollo and collaborators concluded that most species of cephalopods and fish can potentially harbor these marine parasitic nematodes, as 200 fish and 25 cephalopods species have been identified as hosts for *Anisakis* spp. [58], as well as crustacean-some of them of commercial importance [59]. 

The percentage of parasitism of fish by anisakids species may be affected by different factors derived, in part, from the characteristics of their biological cycle, with the host and its geographical origin having the greatest influence [60,61,62,63]. Several authors have considered that the differences observed between the different geographical areas are due to the number of cetaceans that live in each area [9,63,64]. In the case of the Canary Islands, as we mentioned above, the number of cetaceans is high, and consequently, the number of *Anisakis* would also have to be elevated, since the probability of these completing their cycle is high. The prevalence data observed in some of the families analyzed, such as the Merlucciidae family (85.18%), are similar to the prevalence rates reported worldwide [65]. In this sense, several articles have shown that the fish families most vulnerable to *Anisakis* infection were Merluccidae, Lophiidae, Trichiuridae, Zeidae and Gadidae [65].

Spain is the fourth country in the world in terms of fish consumption, with an average of 46.5 kg per person, ranking behind Portugal, Norway and Japan, according to official figures of the Food and Agriculture Organization of the United Nations (FAO) [66]. In the Canary Islands, the consumption of fish is common, and several typical dishes of the local cuisine are based on fish products, such as “jareas”, consisting of gutted fish, which are dehydrated with salt and dried in the sun for about four days. In addition, the production of canned seafood is common in some of the coastal regions of the archipelago, as is the consumption of anchovies in vinegar. Among the most consumed species in the archipelago are *M. merluccius*, *S. colias* and *S. scombrus* [61], analyzed in this work, in addition to *T. picturatus* and *A. thazard*, also of high consumption. In the present study, *Anisakis* larvae were found in four of these species: *M. merluccius*, *S. colias*, and *S. scombrus* and *A. thazard*. 

Globally, among seafood-borne parasites, members of the genus *Anisakis* are considered the most important parasites in relation to human infections [65], since some of them can cause a disease called anisakiasis [67] of serious public health concern globally, being one of the most severe fish-transmitted infections to humans [68]. They are among a few parasites that are known to be dangerous and deadly, even if they are dead in properly cooked seafood [68]. Some studies report patients reacting to *Anisakis* proteins after the ingestion of cooked or canned fish [69], although it is thought that prior exposure to live larvae is necessary [68]. Many *Anisakis* allergens are resistant to cooking and degradation by the digestive enzyme pepsin. 

Of the 10 accepted *Anisakis* species, 5 were identified molecularly in this study in the Canary coast, two of them, *A. simplex* (s.s.) and *A. pegreffi,* being of health relevance, previously cited as causing anisakiasis. To our knowledge, the present study provides the first nucleotide sequence data of *Anisakis* spp. in *A. thazard, M. merluccius, S. colias* and *S. scombrus* from the Canary coast.

With regard to the two *Anisakis* species with sanitary relevance found in the fish of this study, *A. simplex* (s.s.) and *A. pegreffii* are sibling species, under sympatric conditions, sharing intermediate and paratecnic hosts [62]. Over the years, several authors have studied and compared the pathogenicity of both species, since they are the species involved in cases of anisakiasis in humans. Previous studies have shown that *A. simplex* (s.s) is more pathogenic than *A. pegreffii* due to its greater penetration capacity, greater tolerance to acid and a greater tendency to migrate to the muscles [70,71,72,73]. In Japan, most cases of human anisakiasis, where the identification of molecular species was carried out, are caused by infection by *A. simplex* (s.s.), while in Italy, the responsible species is *A. pegreffii* [67,71,73,74,75,76]. 

The data on anisakidosis in the Canary Islands are scarce compared to those of other regions, since its declaration is not mandatory, and a specific diagnosis is often not made in hospitals. In this study, *A. simplex* (s.s.) was detected in *A. thazard, M. merluccius* and *S. scombrus* with a high prevalence. On the other hand, *A. pegreffii*, was also detected in *A. thazard* and *M. merluccius*. These fish species are highly consumed in the Canary Islands and used in a wide variety of typical dishes, so it is important to take this into account when consuming raw or undercooked fish due to the risk of the presence of this nematode.

*Anisakis physiteris* infection has rarely been reported in some pelagic and demersal fish species in the Atlantic and Pacific Oceans—concretely, in species of fish of the family Scombridae, *Scomber australasicus, S. japonicus* and *S. scombrus* [62]. In this study, this nematode was detected in *S. colias*, constituting the first detection of *A. physiteris* in this species.

Regarding *A. typica*, previous studies have detected that it presents lower proportion compared to other *Anisakis* species. However, this species has higher proportion in pelagic species of migratory fish, such as *A. thazard,* in Indonesian waters, where they have high prevalence [77]. In our study, *A. typica* was detected only in *A. thazard*, agreeing with the aforementioned antecedents.

Up to now, a small number of teleost species have been identified as intermediate/paratenic hosts of *A. nascettii*, most of them belonging to the demersal teleost species of the families Merlucciidae, Carangidae, Scombridae, Oreosomatidae, Trachichytiidae and Tichiuridae, being rarely cited; in fact, the records for these fish species consist of a very low number of larvae identified among the hundreds of other *Anisakis* species [6,56]. In our study, *A. nascetti* was identified by nucleotide sequence in *S. colias*, previously identified in the Canary Islands by the PCR-RFLP technique [78].

The majority of human reports only identified the parasite as *Anisakis* larval type [79], and even if the parasite was reported as *A. simplex* (s.s.), no reliable evidence was provided to adequately confirm the species’ identity. This, in addition to the fact that in Spain the declaration of human cases of anisakidosis is not obligatory, can be indicative of an underdiagnosis or, directly, a misdiagnosis of cases of this parasitic disease. 

It is important to note that, in this study, the identification of several species of *Anisakis* was carried out with the contribution of nucleotide sequences for the first time in a wide range of fish species caught in the waters of the Canary Islands with a high interest in human consumption. In addition, the prevalence data were provided for these species to be taken into account when consuming them. The species-level identification of these parasitic nematodes has sanitary relevance, since, depending on the species, the associated pathology will be different. 

## 5. Conclusions

In the present study, five out of the ten accepted *Anisakis* species were detected in commercial fish caught of the Canary coast, two of them being zoonotic species, *A. simplex* (s.s.) and *A. pegreffii*. Moreover, a high prevalence of anisakids larvae was detected in *A. thazard, M. mercluccius, S. colias* and *S. scombrus*, the species being in high demand from consumers in the Canary Islands, increasing the risk for public health by the consumption of raw or undercooked fish. It would be interesting to conduct surveillance studies on anisakids in other fish species, with an interest in consumption in the Canary Islands, to know their health status and take it into account when consuming and assess its possible impact on public health. Finally, the implementation of diagnosis at the species level in hospitals would be interesting.

## Figures and Tables

**Table 1:** Record of measurements, weight and body length of fish examined during October 2020–December 2021. Prevalence, mean intensity and mean abundance of *Anisakis* larvae found.

Host Species (Common Name)	Prevalence of *Anisakis* Larvae P (%) (+/n) (CI 95%)	N_coll_	Mean Intensity (CI 95%)	Mean Abundance (CI 95%)	Mean T.W. ± S.D (CI 95%)	Mean T.L. ± S.D (CI 95%)
*Auxis thazard* (frigate mackerel)	21.43% (3/14) (4.66%, 50.8%)	14	4.67 (1.00, 7.67)	1.00 (0.07, 3.62)	340.18 ± 29.97 (322.88, 357.49)	29.24 ± 0.87 (26.14, 28.97)
*Diplodus sargus sargus* (white seabream)	0% (0/2) -	0	-	-	257.745 ± 55.22 -	25.50 ± 2.12 -
*Merluccius merluccius* (European hake)	85.18% (23/27) (66.27%, 95.81%)	205	8.91 (5.44, 16.76)	7.59 (4.44, 14.45)	271.69 ± 57.34 (249.01, 294.38)	27.56 ± 3.58 (26.24, 28.86)
*Mullus surmuletus* (red mullet)	0% (0/6) -	0	-	-	219.07 ± 61.92 (190.74, 295.3)	23.30 ± 0.84 (22.4, 23.8)
*Pagellus erythinus* (common Pandora)	0% (0/11) -	0	-	-	222.06 ± 69.04 (190.03, 270.69)	25.59 ± 2.55 (24.41, 27.27)
*Sardinella aurita* (sardinelle)	0% (0/21) -	0	-	-	117.78 ± 22.00 (108.66, 126.89)	21.16 ± 2.37 (20.08, 22.24)
*Sarpa salpa* (salema)	0% (0/5) -	0	-	-	269.90 ± 26.83 (251.57, 295.93)	26.90 ± 0.74 (26.3, 27.4)
*Scomber colias* (Atlantic chub mackerel)	26.6% (8/30) (12.28%, 45.89%)	10	1.25 (1.00, 1.50)	0.33 (0.13, 0.57)	237.73 ± 88.67 (210.05, 274.06)	29.99 ± 3.58 (28.65, 31.32)
*Scomber scombrus* (mackerel)	62.5% (10/16) (35.43%, 84.8%)	266	26.60 (8.80, 56.65)	16.63 (5.12, 39.25)	330.96 ± 51.37 (308.65, 357.14)	29.95 ± 2.81 (28.93, 31.84)
*Serranus atricauda* (black tail comber)	0% (0/6)	0	-	-	138.36 ± 19.27 (124.37, 152.25)	22.08 ± 1.02 (21.33, 22.75)
*Trachurus picturatus* (blue jack mackerel)	0% (0/34)	0	-	-	52.90 ± 13.10 (49.19, 57.87)	18.69 ± 1.36 (18.22, 19.17)

N_coll_: larvae collected; CI: Confidence interval of 95%; Mean T.W. ± S.D (min, max) (g): average total weight with standard deviation and (minimum value-maximum value) obtained; Mean T.L. ± S.D (min, max) (cm): average total body length with standard deviation and (minimum value-maximum value) obtained; Prevalence of *Anisakis* larvae P (%) (+/n); Mean intensity % (range); Mean abundance % (range).

**Table 2:** List of species of fish infected and *Anisakis* species identified.

Host Species	Molecularly Identified Species of *Anisakis*
*Auxis thazard*	*Anisakis simplex* (s.s.) *Anisakis pegreffii* *Anisakis typica* *Anisakis simplex* × *Anisakis pegreffii*
*Merluccius merluccius*	*Anisakis simplex* (s.s.) *Anisakis pegreffii* *Anisakis simplex* × *Anisakis pegreffii*
*Scomber colias*	*Anisakis nascettii* *Anisakis physeteris*
*Scomber scombrus*	*Anisakis simplex* (s.s.)

## Data Availability

Not applicable.

## Supplementary Materials

The following supporting information can be downloaded at: https://www.mdpi.com/article/10.3390/ani12192634/s1, Table S1: GenBank accession numbers of *Anisakis* larvae obtained from commercial fishes from the Canary Islands coast (Spain), examined during October 2020–December 2021.

## References

[1] Smith H.H., Wootten R. (1978). *Anisakis* and anisakiasis. Adv. Parasitol..

[2] Bao M., Pierce G.J., Pascual S., González-Muñoz M., Mattiucci S., Mladineo I., Cipriani P., Bušelić I., Strachan N.J. (2017). Assessing the risk of an emerging zoonosis of worldwide concern: Anisakiasis. Sci. Rep..

[3] Lymbery A.J., Cheah F.Y. (2007). Anisakid nematodes and anisakiasis. Food-Borne Parasitic Zoonoses.

[4] Chai J.Y., Murrell K.D., Lymbery A.J. (2005). Fish-borne parasitic zoonoses: Status and issues. Int. J. Parasitol..

[5] Klimpel S., Palm H.W. (2011). Anisakid nematode (Ascaridoidea) life cycles and distribution: Increasing zoonotic potential in the time of climate change?. Progress in Parasitology.

[6] Mattiucci S., Nascetti G. (2008). Advances and trends in the molecular systematics of anisakid nematodes, with implications for their evolutionary ecology and host parasite co-evolutionary processes. Adv. Parasitol..

[7] Mattiucci S., Paoletti M., Webb S.C. (2009). *Anisakis nascettii* n. sp. (Nematoda: Anisakidae) from beaked whales of the southern hemisphere: Morphological description, genetic relationships between congeners and ecological data. Syst. Parasitol..

[8] Cavallero S., Martini A., Migliara G., De Vito C., Iavicoli S., D’Amelio S. (2018). Anisakiasis in Italy: Analysis of hospital discharge records in the years 2005–2015. PLoS ONE.

[9] Carrascosa M.F., Mones J.C., Salcines-Caviedes J.R., Román J.G. (2015). A man with unsuspected marine eosinophilic gastritis. Lancet Infect. Dis..

[10] Pons F.R., Beltran J.G., Gonzalez R.A., Gonzalez M.A.Á., Cusco J.M.D., Priego L.B., Urgorri A.S. (2015). An unusual presentation of anisakiasis in the colon (with video). Gastrointest. Endos..

[11] Van Thiel P.H., Kuipers F.C., Roskam R.T. (1960). A nematode parasitic to herring, causing acute abdominal syndromes in man. Trop. Geogr. Med..

[12] Plath F., Holle A., Zendeh D., Möller F.W., Barten M., Reisinger E.C., Liebe S. (2001). Anisakiasis of the stomach—A case report from Germany. Z. Gastroenterol..

[13] Mattiucci S., Fazii P., De Rosa A., Paoletti M., Megna A.S., Glielmo A., De Angelis M., Costa A., Meucci C., Calvaruso V. (2013). Anisakiasis and gastroallergic reactions associated with *Anisakis pegreffii* infection, Italy. Emerg. Infect. Dis..

[14] Mumoli N., Merlo A. (2013). Colonic anisakiasis. CMAJ..

[15] Mladineo I., Trumbić Ž., Radonić I., Vrbatović A., Hrabar J., Bušelić I. (2017). *Anisakis simplex* complex: Ecological significance of recombinant genotypes in an allopatric area of the Adriatic Sea inferred by genome-derived simple sequence repeats. Int. J. Parasitol..

[16] Dupouy-Camet J., Touabet-Azouzi N., Fréalle E., Van Cauteren D., Yera H., Moneret-Vautrin A. (2016). Incidence de l’anisakidose en France. Enquête rétrospective 2010–2014. Bull. Epidemiol. Hebd..

[17] Lucas S.B., Cruse J.P., Lewis A.A.M. (1985). Anisakiasis in the United Kingdom. Lancet..

[18] Cocheton J.J., Cabou I., Lecomte I. (1991). Anisakiase et infections par les Anisakidés. Ann. Med. Interne.

[19] Eiras J.C., Pavanelli G.C., Takemoto R.M., Nawa Y. (2018). Fish-borne nematodiases in South America: Neglected emerging diseases. J. Helminthol..

[20] Hiramoto J.T., Tokeshi J. (1991). Anisakiasis in Hawaii: A radiological diagnosis. Hawaii J. Med. Public. Health..

[21] Couture C., Gagnon J., Desbiens C. (2003). Human intestinal anisakiosis due to consumption of raw salmon. Am. J. Surg. Pathol..

[22] Audicana M.T., Kennedy M.W. (2008). *Anisakis simplex*: From obscure infectious worm to inducer of immune hypersensitivity. Clin. Microbiol. Rev..

[23] Li S.W., Shiao S.H., Weng S.C., Liu T.H., Su K.E., Chen C.C. (2015). A case of human infection with *Anisakis simplex* in Taiwan. Gastrointest. Endosc..

[24] Sohn W.M., Na B.K., Kim T.H., Park T.J. (2015). Anisakiasis: Report of 15 gastric cases caused by *Anisakis* type I larvae and a brief review of Korean anisakiasis cases. Korean. J. Parasitol..

[25] Paltridge G.P., Faoagali J.L., Angus H.B. (1984). Intestinal anisakiasis: A new New Zealand disease. N. Z. Med. J..

[26] EFSA Panel on Biological Hazards (BIOHAZ) (2010). Scientific opinion on risk assessment of parasites in fishery products. EFSA.

[27] Leuckart R. (1876). Die menschlichen Parasiten und die von ihren herriihrend Krankheiten. Leipzig.

[28] Van Thiel P.H. (1962). Anisakiasis. Parasitology.

[29] Villazanakretzer D.L., Napolitano P.G., Cumming K.F., Magann E.F. (2016). Fish parasites a growing concern during pregnancy. Obstet. Gynecol. Surv..

[30] Moore David A.J., Girdwood R.W.A., Chiodini Peter L. (2002). Treatment of anisakiasis with albendazole. Lancet.

[31] Dziekonska-Rynko J., Rokicki J., Jablonowski Z. (2002). Effects of ivermectin and albendazole against *Anisakis simplex* in vitro and in guinea pigs. J. Parasitol..

[32] Arias Diaz J., Zuloaga J., Vara E., Balibrea J., Balibrea J.L. (2006). Efficacy of albendazole against *Anisakis simplex* larvae in vitro. Dig. Liver Dis..

[33] McCarthy J., Moore T.A. (2000). Emerging helminth zoonoses. Int. J. Parasitol..

[34] Reglamento (CE) Nº 854/2004 del Parlamento Europeo y del Consejo, de 29 de Abril de 2004. https://www.boe.es/buscar/doc.php?id=DOUE-L-2004-81037.

[35] Berland B. (1961). Nematodes from some Norwegian marine fishes. Sarsia.

[36] Petter A.J., Maillard C. (1988). Larves d’ascarides parasites de poissons en Méditerranée occidentale. Bull. Mus. Natl. Hist. Nat..

[37] Shamsi S., Gasser R., Beveridge I. (2013). Description and genetic characterisation of *Hysterothylacium* (Nematoda: Raphidascarididae) larvae parasitic in Australian marine fishes. Parasitol. Int..

[38] Bush A.O., Lafferty K.D., Lotz J.M., Shostak A.W. (1997). Parasitology meets ecology on its own terms: Margolis et al. revisited. J. Parasitol..

[39] Tibshirani R.J., Efron B. (1993). An introduction to the bootstrap. Monogr. Stat. Appl. Probab..

[40] R Core Team (2019). R: A Language and Environment for Statistical Computing.

[41] López C., Clemente S., Almeida C., Brito A., Hernández M. (2015). A genetic approach to the origin of *Millepora* sp. in the eastern Atlantic. Coral Reefs..

[42] Zhu X., Gasser R.B., Podolska M., Chilton N.B. (1998). Characterisation of anisakid nematodes with zoonotic potential by nuclear ribosomal DNA sequences. Int. J. Parasitol..

[43] Nadler S.A., Hudspeth D.S.S. (2000). Phylogeny of the Ascaridoidea (Nematoda: Ascaridida) based on three genes and morphology: Hypotheses of structural and sequence evolution. J. Parasitol..

[44] Kumar S., Stecher G., Li M., Knyaz C., Tamura K. (2018). MEGA X: Molecular evolutionary genetics analysis across computing platforms. Mol. Biol. Evol..

[45] Saitou N., Nei M. (1987). The neighbor-joining method: A new method for reconstructing phylogenetic trees. Mol. Biol. Evol..

[46] Tamura K., Nei M. (1993). Estimation of the number of nucleotide substitutions in the control region of mitochondrial DNA in humans and chimpanzees. Mol. Biol. Evol..

[47] Bas C., Castro J.J., Hernández-García V., Lorenzo J.M., Moreno T., Pajuelo J.G., Ramos A.J. (1995). La pesca en Canarias y áreas de influencia.

[48] Aristegui J., Barton E.D., Álvarez-Salgado X.A., Santos A.M.P., Figueiras F.G. (2009). Subregion al ecosystem variability in the Canary Current upwelling. Prog. Oceanogr..

[49] Aristegui J., Sangrá P., Hernández-León S., Cantón M., Hernández-Guerra A., Kerling J.L. (1994). Island-induced eddies in the Canary Islands. Depp-Sea Res. I Oceanog. Res. Pap..

[50] Sangrà P., Pascual A., Rodríguez-Santana Á., Machín F., Mason E., McWilliams J.C., Auladell M. (2009). The Canary Eddy Corridor: A major pathway for long-lived eddies in the subtropical North Atlantic. Depp-Sea Res. I Oceanog. Res. Pap..

[51] Lobel P.S., Robinson A.R. (1986). Transport and entrapment of fish larvae by ocean mesoscale eddies and currents in Hawaiian waters. Deep Sea Res..

[52] Lobel P.S., Robinson A.R. (1988). Larval fishes and zooplankton in cyclonic eddy in Hawaiian waters. J. Plankton Res..

[53] Crawford R., Jorgenson J. (1990). Density distribution of fish in the presence of whales at the Admiralty Inlet land fast ice edge. Arctic.

[54] Gómez M. (1991). Biomasa y Actividad Metabólica del Zooplancton en Relación con un Efecto de Masa de isla en Aguas de Gran Canaria.

[55] Rodríguez J.M., Barton E.D., Hernández-León S., Arístegui J. (2004). The influence of mesoscale physical processes on the larval fish community in the Canaries CTZ, in summer. Prog. Oceanogr..

[56] Moro L., Martín J.L., Garrido M.J., Izquiero I. (2003). Lista de Especies Marinas de Canarias (Algas, Hongos, Plantas y Animales).

[57] Nagasawa K., Moravec F. (1995). Larval anisakid nematodes of Japanese common squid (*Todarodes pacificus*) from the Sea of Japan. J. Parasitol..

[58] Abollo E., Gestal C., Pascual S. (2001). *Anisakis* infestation in marine fish and cephalopods from Galician waters: An updated perspective. Parasitol. Res..

[59] Vidaček S., de las Heras C., Solas M.T., Mendizábal A., Rodriguez-Mahillo A.I., González-Muñoz M., Tejada M. (2009). *Anisakis simplex* allergens remain active after conventional or microwave heating and pepsin treatments of chilled and frozen L3 larvae. J. Sci. Food Agric..

[60] Cipriani P., Sbaraglia G.L., Palomba M., Giulietti L., Bellisario B., Bušelić I., Mladineo I., Cheleschi R., Nascetti G., Mattiucci S. (2018). *Anisakis pegreffii* (Nematoda: Anisakidae) in European anchovy *Engraulis encrasicolus* from the Mediterranean Sea: Fishing ground as a predictor of parasite distribution. Fish. Res..

[61] Gutiérrez-Galindo J.F., Osanz-Mur A.C., Mora-Ventura M.T. (2010). Occurrence and infection dynamics of anisakid larvae in *Scomber scombrus*, *Trachurus trachurus*, *Sardina pilchardus*, and *Engraulis encrasicolus* from Tarragona (NE Spain). Food Control.

[62] Mattiucci S., Cipriani P., Levsen A., Paoletti M., Nascetti G. (2018). Molecular epidemiology of *Anisakis* and Anisakiasis: An ecological and evolutionary road map. Adv. Parasitol..

[63] Rello F.J., Adroher F.J., Benítez R., Valero A. (2009). The fishing area as a possible indicator of the infection by anisakids in anchovies (*Engraulis encrasicolus*) from southwestern Europe. Int. J. Food Microbiol..

[64] Klapper R., Kuhn T., Münster J., Levsen A., Karl H., Klimpel S. (2015). Anisakid nematodes in beaked redfish (*Sebastes mentella*) from three fishing grounds in the North Atlantic, with special notes on distribution in the fish musculature. Vet. Parasitol..

[65] Rahmati A.R., Kiani B., Afshari A.M., Moghaddas E., Williams M., Shamsi S. (2020). World-wide prevalence of *Anisakis* larvae in fish and its relationship to human allergic anisakiasis: A systematic review. Parasitol. Res..

[66] FAO Organización de las Naciones Unidas para la Alimentación Agricultura y la Agricultura Alimentación, Fishery and Aquaculture Statistics—Estadísticas de Pesca y Acuicultura 2021. https://www.fao.org/statistics/es/.

[67] D’Amelio S., Mathiopoulos K.D., Brandonisio O., Lucarelli G., Doronzo F., Paggi L. (1999). Diagnosis of a case of gastric anisakidosis by PCR-based restriction fragment length polymorphism analysis. Parassitologia.

[68] Audicana M.T., Ansotegui I.J., Fernandez de Corres L., Kennedy M.W. (2002). *Anisakis simplex*: Dangerous—Dead and alive?. Trends Parasitol..

[69] Del Pozo M.D., Audicana M., Diez J.M., Muñoz D., Ansotegui I.J., Fernández E., García M., Etxenagusia M., Moneo I., Fernández de Corres L. (1997). *Anisakis simplex*, a relevant etiologic factor in acute urticaria. Allergy.

[70] Suzuki J., Murata R., Hosaka M., Araki J. (2010). Risk factors for human Anisakis infection and association between the geographic origins of *Scomber japonicus* and anisakid nematodes. Int. J. Food Microbiol..

[71] Arizono N., Yamada M., Tegoshi T., Yoshikawa M. (2012). *Anisakis simplex* sensu stricto and *Anisakis pegreffii*: Biologicalcharacteristics and pathogenetic potential in human anisakiasis. Foodborne Pathog. Dis..

[72] Jeon C.H., Kim J.H. (2015). Pathogenic potential of two sibling species, *Anisakis simplex* (ss) and *Anisakis pegreffii* (Nematoda: Anisakidae): In Vitro and in vivo studies. BioMed Res. Int..

[73] Umehara A., Kawakami Y., Araki J., Uchida A. (2007). Molecular identification of the etiological agent of the human anisakiasis in Japan. Parasitol. Int..

[74] Mattiucci S., Abaunza P., Damiano S., Garcia A., Santos M.N., Nascetti G. (2007). Distribution of Anisakis larvae, identified by genetic markers, and their use for stock characterization of demersal and pelagic fish from European waters: An update. J. Helminthol..

[75] Mattiucci S., Paoletti M., Borrini F., Palumbo M., Palmieri R.M., Gomes V., Nascetti G. (2011). First molecular identification of the zoonotic parasite *Anisakis pegreffii* (Nematoda: Anisakidae) in a paraffin-embedded granuloma taken from a case of human intestinal anisakiasis in Italy. BMC. Infect. Dis..

[76] Fumarola L., Monno R., Ierardi E., Rizzo G., Giannelli G., Lalle M., Pozio E. (2009). *Anisakis pegreffi* etiological agent of gastric infections in two Italian women. Foodborne Pathog. Dis..

[77] Palma R., Mattiucci S., Panetta C., Raniolo M., Magliocca F.M., Pontone S. (2018). Paucisymptomatic gastric anisakiasis: Endoscopical removal of *Anisakis* sp. larva. Minim. Invasive Surg..

[78] Costa G., Cavallero S., D’Amelio S., Paggi L., Santamaria M., Perera C., Khadem M. (2011). Helminth parasites of the Atlantic chub mackerel, *Scomber colias* Gmelin, 1789 from Canary Islands, Central North Atlantic, with comments on their relations with other Atlantic regions. Acta Parasitol..

[79] Dupouy-Camet J., Bruschi F. (2014). Helminth Infections and Their Impact on Global Public Health.

