# Supplementary material for: Morphological and Molecular Identification of Anisakis spp. (Nematoda: Anisakidae) in Commercial Fish from the Canary Islands Coast (Spain): Epidemiological Data
Source: Animals (Basel). 2022 Sep 30;12(19):2634. doi: 10.3390/ani12192634 (PMC9559264; doi:10.3390/ani12192634)
Supplement: Supplementary file 1 [file animals-12-02634-s001.zip › animals-1861707-supplementary.pdf]

Table S1. GenBank accession numbers of *Anisakis* larvae obtained from commercial fishes from the Canary Islands coast (Spain), examined during October 2020- December 2021.

***Aniskais* larva ID**	**Fish species**	**ITS1**	**mtDNA cox2**	**GenBank Access numbers**
Y5	*Scomber scombrus*	*Anisakis simplex* (s.s.)	*Anisakis simplex* (s.s.)	ITS1: OM328182 mtDNA *cox*2: OM417318
Y32	*Scomber scombrus*	*Anisakis simplex* (s.s.)	*Anisakis simplex* (s.s.)	ITS1: OM328167 mtDNA *cox*2: OM417320
Y47	*Merluccius merluccius*	*Anisakis pegreffii*	*Anisakis pegreffii*	ITS1: OM328168 mtDNA *cox*2: OM417321
Y50	*Merluccius merluccius*	*Anisakis pegreffii*	*Anisakis pegreffii*	ITS1: OM328181 mtDNA *cox*2: OM417323
Y70	*Merluccius merluccius*	*Anisakis pegreffii*	*Anisakis pegreffii*	ITS1: OM328183 mtDNA *cox*2: OM417322
RANi26	*Merluccius merluccius*	*Anisakis simplex* (s.s.)	*Anisakis simplex* (s.s.)	ITS1: OM328178 mtDNA *cox*2: OM417311
RANi29	*Merluccius merluccius*	*Anisakis simplex* (s.s.)	*Anisakis simplex x Anisakis pegreffii*	ITS1: OM328179 mtDNA *cox*2: OM417312
RANi30	*Scomber colias*	*Anisakis physeteris*	No sequence obtained	ITS1: OM328180
RANi34	*Scomber colias*	*Anisakis nascettii*	*Anisakis nascettii*	ITS1: OM328166 mtDNA *cox*2: OM417315
RANi100	*Auxis thazard*	*Anisakis simplex* (s.s.)	*Anisakis simplex* (s.s.)	ITS1: OM328160 mtDNA *cox*2: OM417314
RANi101	*Auxis thazard*	*Anisakis simplex* (s.s.)	*Anisakis simplex x Anisakis pegreffii*	ITS1: OM328161 mtDNA *cox*2: OM417313
RANi102	*Auxis thazard*	*Anisakis simplex* (s.s.)	*Anisakis simplex* (s.s.)	ITS1: OM328162 mtDNA *cox*2: OM417319
RANi103	*Auxis thazard*	*Anisakis simplex* (s.s.)	*Anisakis simplex* (s.s.)	ITS1: OM328163 mtDNA *cox*2: OM417316
RANi105	*Auxis thazard*	*Anisakis pegreffii*	*Anisakis pegreffii*	ITS1: OM328164 mtDNA *cox*2: OM417325
RANi106	*Auxis thazard*	*Anisakis simplex* (s.s.)	*Anisakis simplex* (s.s.)	ITS1: OM328159 mtDNA *cox*2: OM417317
RANi107	*Auxis thazard*	*Anisakis typica*	*Anisakis typica*	ITS1: OM328165 mtDNA *cox*2: OM417324
